# Role of chromatin and transcriptional co-regulators in mediating p63-genome interactions in keratinocytes

**DOI:** 10.1186/1471-2164-15-1042

**Published:** 2014-11-29

**Authors:** Isha Sethi, Satrajit Sinha, Michael J Buck

**Affiliations:** Department of Biochemistry and Center of Excellence in Bioinformatics and Life Sciences, State University of New York at Buffalo, Buffalo, USA

**Keywords:** p63, Chromatin, ChIP-Seq, Transcription, Keratinocyte, ENCODE

## Abstract

**Background:**

The Transcription Factor (TF) p63 is a master regulator of epidermal development and differentiation as evident from the remarkable skin phenotype of p63 mouse knockouts. Furthermore, ectopic expression of p63 alone is sufficient to convert simple epithelium into stratified epithelial tissues *in vivo* and p63 is required for efficient transdifferentiation of fibroblasts into keratinocytes. However, little is known about the molecular mechanisms of p63 function, in particular how it selects its target sites in the genome. p63, which acts both as an activator and repressor of transcription, recognizes a canonical binding motif that occurs over 1 million times in the human genome. But, in human keratinocytes less than 12,000 of these sites are bound *in vivo* suggesting that underlying chromatin architecture and cooperating TFs mediate p63-genome interactions.

**Results:**

We find that the chromatin architecture at p63-bound targets possess distinctive features and can be used to categorize p63 targets into proximal promoters (1%), enhancers (59%) and repressed or inactive (40%) regulatory elements. Our analysis shows that the chromatin modifications H3K4me1, H3K27me3, along with overall chromatin accessibility status can accurately predict bonafide p63-bound sites without a priori DNA sequence information. Interestingly, however there exists a qualitative correlation between the p63 binding motif and accessibility and H3K4me1 levels. Furthermore, we use a comprehensive *in silico* approach that leverages ENCODE data to identify several known TFs such as AP1, AP2 and novel TFs (RFX5 for e.g.) that can potentially cooperate with p63 to modulate its myriad biological functions in keratinocytes.

**Conclusions:**

Our analysis shows that p63 bound genomic locations in keratinocytes are accessible, marked by active histone modifications, and co-targeted by other developmentally important transcriptional regulators. Collectively, our results suggest that p63 might actively remodel and/or influence chromatin dynamics at its target sites and in the process dictate its own DNA binding and possibly that of adjacent TFs.

**Electronic supplementary material:**

The online version of this article (doi:10.1186/1471-2164-15-1042) contains supplementary material, which is available to authorized users.

## Background

Tp63 is an important transcription factor of the p53/p63/p73 family that dictates a wide range of cellular properties including but not limited to stem cell renewal, lineage choices and maintaining the balance between proliferation and differentiation [1, 2]. This diverse function of p63 is critical for morphogenesis during development, particularly for epithelial-enriched tissues such as the skin and its appendages such as the hair follicles and mammary glands. Indeed, p63-null mice die after birth and exhibit a dramatic agenesis of epithelial-rich structures and widespread developmental defects of the limb, orofacial region, and external genitalia [3, 4]. These p63-deficient structural defects are thought to be the result of a failed program of epithelial stratification and/or diminished capacity for stem cell renewal, both of which can jeopardize normal epithelial-stromal interactions needed during embryonic organ development [5, 6]. In agreement with the mouse phenotype, p63 mutations in humans lead to congenital abnormalities such as abnormal limb development and ectodermal dysplasia, which are associated with a spectrum of developmental disorders including AEC or EEC syndrome [7, 8].

The biological function of p63 is mediated by several isoforms derived from distinct transcripts [1]. These include the longer TAp63 isoforms and N-terminal deleted ΔNp63 isoforms generated from an internal promoter located within intron 3. Furthermore alternative splicing can result in α, β, and γ isoforms, which differ in the C-terminus. All p63 isoforms share the DNA-binding and oligomerization domains, which are analogous to that of p53. It is now well-established that ∆Np63, especially ∆Np63α is the predominant isoform that is present in most epithelial cells such as the keratinocytes of the skin [9]. Importantly both gene complementation studies and isoform specific knockouts have conclusively affirmed that ∆Np63 harbors most of the function and biological activity of p63, particularly as it pertains to the epithelial tissues [10–14].

The role of p63 in regulating transcription during development has been extensively studied in skin where ∆Np63 is highly expressed and regulates the transition from simple ectodermal cells to stratified epithelium [5, 15]. Given the master regulatory function of p63, it is not surprising that the repertoire of p63-targets is vast and represents practically every crucial gene regulatory and signaling pathway. This is evident from the ~11,000 binding sites for p63 in human keratinocytes as determined by chromatin immunoprecipitation with next-generation sequencing (ChIP-seq) studies [16]. p63 controls expression of basal keratin genes K5 and K14 and regulates MYC levels thereby controlling keratinocyte proliferation via the Wnt/β-catenin and Notch signaling pathways [10, 17, 18]. The keratinocyte differentiation program is also regulated by p63, in part via its effect on the ZNF750-KLF4 regulatory axis [19].

While the identification of p63 bound *cis*-regulatory elements in keratinocytes has received much attention, the mechanics of p63-DNA interaction is still relatively unknown. p63 binds a canonical motif, defined as closely spaced 2 decamers (RRRCRWGYYY, RRRCWYGYYY), although there is growing evidence that p63 can target sites that do not completely conform to this consensus sequence, including half sites [20]. Given the degenerate nature of the p63 binding motif, it is not surprising that by conservative estimates, there are more than 1 million such potential sites in the human genome. However, as is the case with most other Transcription Factors (TFs), only a small subset of these sites are bound by p63 *in vivo*[16, 21]. It is likely that the local chromatin architecture, among other factors plays an important deterministic role in dictating how and why p63 selects its target DNA. Hence, this is an important area of future investigation; especially given the increasing evidence that p63 can play an important role in modulating the chromatin structure. Indeed, recent studies have demonstrated that p63 can functionally interact with several epigenetic factors in keratinocytes, which can in turn profoundly influence p63-dependent transcriptional activation and repression. Examples of such interactions include the reinforcement of p63 mediated repression of p16 by Lsh, a member of the SNF2 family of chromatin remodeling ATPases [22], direct recruitment of histone deacetylases, HDAC1 and HDAC2 by ∆Np63 during repression of target genes in the embryonic epidermis [23] and the crosstalk between p63 and chromatin organizer Satb1 in regulating keratinocyte differentiation genes [24]. p63 can also control higher-order chromatin structure in epidermal progenitor cells during skin development by regulating Brg1, a ATP-dependent chromatin remodeler [25]. Given these emerging links between p63 and chromatin, it is important that any comprehensive studies on the mechanism of p63-genome interactions takes into account the underlying state of epigenetic modifications.

Here we have utilized the p63 ChIP-Seq dataset and available chromatin modification datasets for Normal Human Epidermal Keratinocytes (NHEK) to investigate the rules that govern binding of p63 to its target DNA. We find that p63 binds to a canonical motif (2 decamers with zero spacer in-between) at the majority (73.3%) of its sites, whereas non-canonical motifs containing 1–15 spacer between decamers are present in only 16.4% of the sites. The chromatin at p63 binding sites is largely marked by active histone modifications (H3K4me1 or H3K4me3 and H3K27ac). Moreover, chromatin accessibility with H3K4me1 can accurately predict bona-fide bound p63 sites without the need for any additional DNA sequence information. Finally, using a comprehensive *in silico* approach, we identify several cooperating TFs that appear to define specific classes of p63 regulated genes.

## Results

### Underlying sequence patterns and chromatin architecture of p63 targets

Several groups have determined global p63 binding locations in various primary and immortalized keratinocytes using ChIP-chip or ChIP-Seq techniques [16, 21, 26–28]. For our studies, we focused on the most comprehensive p63 ChIP-Seq data [16] available to date. It had an added benefit of being generated from primary keratinocytes (NHEK) and more importantly conforming to ENCODE guidelines [29]. To facilitate uniform comparisons across other ENCODE datasets, we re-aligned the p63 ChIP-Seq to the latest human genome build (hg19) with Bowtie [30]. In strong agreement with Kouwenhoven et al., by using high stringency conditions (p-value: 1e^-10^), we identified a reliable and robust dataset of 11632 p63 binding sites that were common among the three biological replicates. On examining the underlying DNA sequence of these p63-ChIPed elements, we found that 73.3% of these sites have at least one p63 canonical motif. Among these, 32% show a close match to the p63 consensus (strong motif) while the remaining 41.3% are a weaker match (Additional file 1: Figure S1). Both the strong and weak canonical motifs are significantly enriched at the p63 ChIPed regions compared to random genomic regions (P value <1×10^-200^). An additional 16.4% of the binding sites show a close match to the non-canonical p63 motif, which has 1–15 base spacers separating the two half-sites (Additional file 2: Figure S2). Interestingly, 10% of the p63 bound genomic sites do not have a recognizable p63 motif raising the possibility that p63 can perhaps also be recruited to target regulatory elements through indirect mechanisms such as protein-protein interactions. Our analysis also revealed that only a few of the p63 binding sites contained just a half site (Additional file 2: Figure S2).

**Figure 1:**
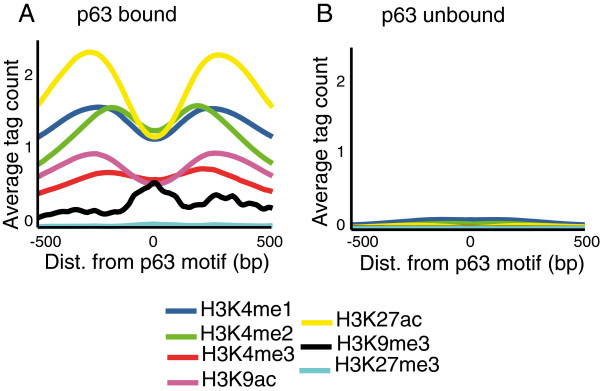
**Chromatin architecture differs between p63 bound and unbound sites.** Average profiles of the 5 active (H3K4me1, H3K4me2, H3K4me3, H3K9ac, H3K27ac) and 2 repressive (H3K9me3, H3K27me3) histone modifications are plotted for a 1 kb window centered at p63 motif. **(A)** Average chromatin architecture at 11632 p63 bound locations. **(B)** Average chromatin architecture at 30,000 unbound locations that have a strong p63 motif. Profiles were generated with ArchTEx [32].

Next we compared the chromatin architecture of the 11,632 p63 bound sites and randomly selected 30,000 unbound sites, which have at least 1 strong p63 canonical motif. For this purpose we focused on 5 active (H3K4me1, H3K4me2, H3K4me3, H3K9ac, H3K27ac) and 2 repressive (H3K9me3, H3K27me3) histone modifications profiles in NHEK cells that have been generated by the ENCODE consortium [31]. As shown in Figure 1A, the genomic segments of DNA bound by p63 are characterized by a chromatin architecture consisting of high H3K27ac, H3K4me1, H3K4me2, and H3K4me3 and low H3K9me3 and H3K27me3 modifications. In contrast, the randomly selected stretches of DNA corresponding to the unbound p63 sites are completely lacking in these chromatin profiles (Figure 1B). Hence chromatin architecture could contribute to selective targeting of p63.

p63 has been shown to target different types of regulatory regions (such as promoters and enhancers) and involved in both activation and repression of gene expression [33–36]. Recent studies have demonstrated that specific states of chromatin modifications at the regulatory regions are strongly associated with the level of gene expression for the corresponding genes [37]. To determine whether such differences exists for distinct classes (active vs. repressed for e.g.) of p63 targets, we clustered the p63 bound regulatory regions by their underlying chromatin architecture. We first divided p63 targets into two groups (Cluster A and B) based on the magnitude of signal (chromatin state) and then performed unsupervised clustering using the spatial arrangement of the histone modifications (chromatin architecture) [38].

**Figure 2:**
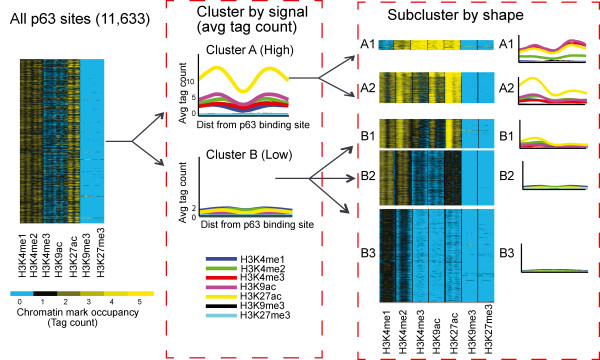
**Clustering of p63 targets by chromatin intensity and shape.** p63 bound locations are clustered by 5 active (H3K4me1, H3K4me2, H3K4me3, H3K9ac, H3K27ac) and 2 repressive (H3K9me3, H3K27me3) histone modifications sequenced in NHEK (Normal Human Epidermal Keratinocytes) cell-line. They are first clustered by average signal intensity across a 1 kb window centered at p63 binding site and then clustered by spatial arrangement of the histone modifications.

Using standard K-means clustering (with K = 4) we were able to capture all patterns within cluster A and B but this resulted in redundancy (i.e. clusters with similar chromatin patterns or mirror images of each other). Therefore, we compared all 8 sub-clusters to each other in both orientations, grouping together those with a Pearson correlation above 0.9. This resulted in the classification of p63 targets into 5 distinct, non-redundant groups (Figure 2 and Additional file 3: Table S1). Based on chromatin based segmentation [39], the majority of p63 targets are predicted to represent enhancers (58.7%) (sub-clusters A2,B1,B2) while only 167 (1.4%) represent active promoters (sub-cluster A1). The distance of the regulatory regions to the nearest TSS supports these predictions (Table 1). It is interesting to note that none of the clusters are characterized by repressive histone marks. However this does not imply that p63 cannot act as a repressor. In fact the largest sub-cluster B3 (39.8% of p63 targets) has very low signal for active chromatin marks and predicted by chromatin based segmentation likely to represent repressed/inactive targets. Indeed, upon probing the RNA-Seq data from keratinocytes, it is evident that the regulatory regions of the B3 sub-cluster are associated with genes that are weakly expressed (Additional file 4: Figure S3). Therefore this cluster might represent repressed/poised genes for which p63 may not play an active role in activation at least in the basal growth conditions of NHEK cells.

**Table 1:** **Annotating p63 targets clustered by chromatin profiles**

Cluster	Size of cluster	Chromatin based segmentation	Median distance to nearest TSS	Median expression of nearest gene (RPKM)	Top GO terms (Biological Process) (Binomial FDR Q-val)	Top PANTHER pathways (Binomial FDR Q-val)
A1	167	Active Promoters	Proximal (898 bp)	8.09	1. Regulation of programmed cell-death (2.98e-2)	1. P53 pathway (1.27e-3)
A2	1309	Strong Enhancers	Distal (35 kb)	5.03	1. Anti-apoptosis (8.28e-14)	1. Apoptosis signaling pathway (1.94e-9)
					2. Hemi-desmosome assembly (1.166e-10)	2. Integrin signaling pathway (1.67e-7)
					3. Epidermis development (1.85e-9)	3. Interleukin signaling pathway (1.1299e-6)
B1	2035	Strong Enhancers	Distal (37 kb)	3.85	1. Epidermis development (3.29e-9)	1. T cell activation (2.06e-4)
					2. Hair follicle development (3.25e-6)	
					3. Hair cycle (3.88e-6)	
B2	3491	Strong/weak enhancers	Distal (49.3 kb)	2.28	1. Response to mechanical stimulus (9.75e-10)	1. T cell activation (1.9e-8)
					2. Negative regulation of intracellular protein kinase cascade (1.54e-6)	2. VEGF signaling pathway (3.77e-5)
					3. Response to reactive oxygen species (2.6e-6)	
B3	4630	Repressed/Inactive	Distal (57.4 kb)	1.09	Hair cycle (1.19e-9)	No term enriched
					Hair follicle development (1.56e-9)	
					Cellular response to gonadotropin stimulus (4.16e-4)	

p63 bound locations are clustered by 5 active (H3K4me1, H3K4me2, H3K4me3, H3K9ac, H3K27ac) and 2 repressive (H3K9me3, H3K27me3) histone modifications sequenced in NHEK cell-line. The 5 groups are annotated by chromatin based segmentation and median distance to nearest TSS. GREAT is used to find the top 3 GO-BP terms and top 3 PANTHER pathways that enrich for each cluster.

We next used Genomic Regions Enrichment of Annotations Tool (GREAT) [40] to determine whether the five clustered groups of p63 targets could be segregated into distinct classes of genes involved in specific biological pathways. Our analysis revealed that each cluster indeed was overrepresented by genes that were involved in closely related, yet disparate biological activity. For example, cluster B1, which primarily encodes for strong enhancers is enriched for Gene-Ontology Biological Process (GO-BP) of epidermis development, while cluster B3 representing repressed/inactive sites is enriched for GO-BP of hair cycle. This raises the intriguing possibility that p63 might play an important role in actively driving the epidermal developmental processes while keeping the hair cell fate repressed – a notion that is supported by data from prior transgenic mouse studies [41]. The complete annotation for each group of p63 targets is provided in Table 1. Thus, collectively the data obtained by chromatin architecture-dependent clustering of p63 targets allowed us to determine both the active and poised targets of p63 and annotate these to specific biological processes (Additional file 3: Table S1).

### p63 binding can be accurately predicted from chromatin modifications

We next implemented two different statistical approaches to test whether epigenetic modifications can be exclusively utilized to identify functional p63 binding sites. We generated a discriminant and a regression model of p63-binding events based on data from histone modifications as determined by ChIP-Seq and chromatin accessibility as inferred from DNase-Seq and FAIRE-Seq experiments. DNase-Seq and FAIRE-Seq assays generate both distinct and overlapping accessibility information about the genomic landscape [42]. Also the different histone modifications usually correspond to transcriptional regulation in a combinatorial fashion [43]. As these various chromatin features are not mutually independent of each other, we created interaction datasets from each pair of chromatin datasets, such as DNase*FAIRE (DF). These were also used as predictors for our statistical models. For the discriminant approach we generated the model on a training dataset containing 5000 p63 binding sites with 30,000 unbound genomic locations and for the regression approach we generated the model on a training dataset containing 5000 p63 binding sites with 45,000 unbound genomic locations (see Methods). We then validated the models on a test dataset of 56,392 genomic locations (6632 p63-positive sites and 49760 p63-negative sites).

**Figure 3:**
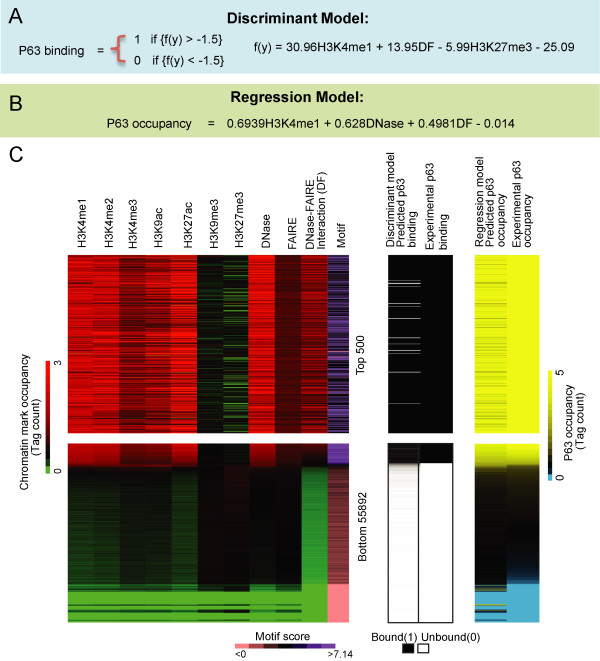
**p63 occupancy can be accurately predicted by chromatin marks.** 5 active (H3K4me1, H3K4me2, H3K4me3, H3K9ac, H3K27ac) and 2 repressive (H3K9me3, H3K27me3) histone modifications with chromatin accessibility as measured by DNase-Seq and FAIRE-Seq were used to construct models of p63 binding. **(A)** Discriminant model for p63 binding. **(B)** Regression model for p63 occupancy. **(C)** The values for 11 datasets used for our models are shown with the data sorted in descending order by experimental p63 occupancy. The standardized number of tags for the histone modifications and chromatin accessibility are displayed. The DNase and FAIRE interaction term (DF) is calculated as product of the two and is displayed in standardized tag space. The quality of the p63 sequence motif was determined by PATSER, good motif matches are purple and low quality motif matches are pink. The results of our discriminant model is compared directly to the experimental determination of binding by MACS. Our regression model is compared directly to experimental occupancy.

The discriminant model identifies boundaries between groups of objects (in this case p63 bound versus unbound sites), the boundaries being defined in terms of those variable characteristics that distinguish the objects into two groups. This technique allows us to determine which chromatin modifications are strongly predictive of p63 binding status (Figure 3A). In addition this model can be used to predict if an unknown site is bound or unbound by p63. We found that a three variable model was able to accurately classify p63 binding sites (sensitivity: 89.8%(±0.3), specificity: 97.6%(±0.02)) (Figure 3C). This model was based on DF, H3K4me1, and H3K27me3 datasets. In this model both DF and H3K4me1 had positive coefficients as anticipated by p63 binding to sites with high values for these chromatin datasets. On the other hand, the repressive modification H3K27me3 had a negative coefficient suggesting that p63 is being excluded from genomic sites with this histone modification.

**Figure 4:**
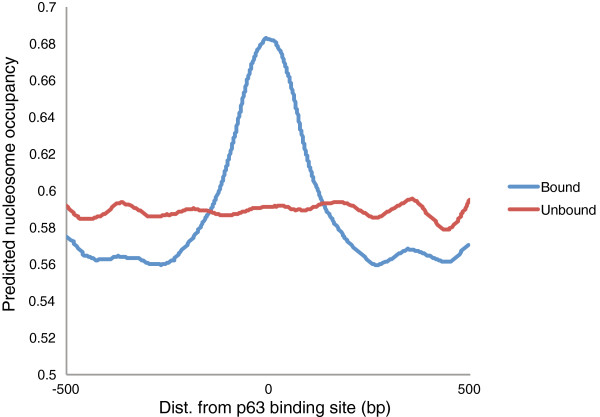
**p63 preferentially binds nucleosome rich regions.** Predicted nucleosome occupancy as calculated by the Nucleosome-DNA interaction model [44], was plotted for a 1 kb window centered at p63 binding site. Blue: Average nucleosome occupancy at 11632 p63 bound locations. Red: Average nucleosome occupancy at 12,000 random unbound locations.

We also utilized the regression model to determine which chromatin characteristic(s) best predicts the occupancy of p63 binding at any genomic location (Figure 3B). When applied to the test dataset, the regression model with only three variables has a MSE (Mean Squared Error) of 0.083(±0.001) and R^2^ of 0.747(±0.011) (P value <1× 10^-200^) (Figure 3C). For the regression model H3K4me1, DNase, and DF variables were the most informative. For both models, a chromatin accessibility dataset (DNase or DF) with H3K4me1 were found to be very predictive (Additional file 5: Table S2, Additional file 6: Table S3). Furthermore, a regression model using only the DF term has a R^2^ = 0.665, which exemplifies the importance of chromatin accessibility in defining true *in vivo* binding sites. However these models do not inform whether the accessibility is a prerequisite or a secondary effect of p63 binding. We therefore integrated the nucleosome-DNA interaction model in our analysis, which is based on the principle that certain DNA sequences show a greater predisposition to wrap around the histone octamer and form nucleosomes [44]. This model has previously been used to predict and experimentally validate that p53 preferentially binds nucleosome rich regions [45]. Our analysis showed that the p63-bound genomic segments in keratinocytes have higher sequence defined nucleosome occupancy than randomly chosen sequences (Figure 4). This suggests that the increased chromatin accessibility at p63 bound sites is likely to be actively shaped by p63-DNA interactions.

**Figure 5:**
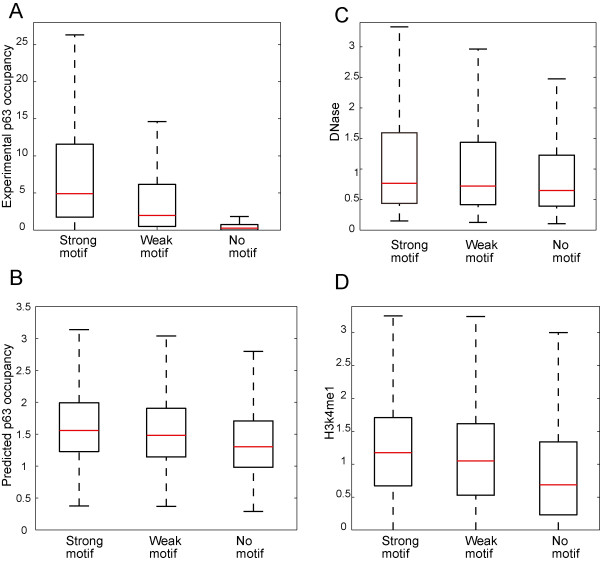
**Significance of p63 motif.** The genomic coordinates of all the accessible locations in human keratinocytes, as determined by DNase-Seq in NHEK cell-line, were obtained from ENCODE via the UCSC genome browser. The 145,203 accessible locations are divided into 3 groups 1) 4153 locations with strong p63 motif (motif score >7.14), 2) 10,503 locations with weak motif (7.14 > motif score >2.24) and 3) 130547 locations with no motif. Box and Whisker Plots are made across 500 bp window for **(A)** Experimental p63 occupancy, **(B)** Predicted p63 occupancy by our best 3 regression model, **(C)** DNase tag density, **(D)** H3K4me1 tag density.

For both the regression and discriminant models, p63 motif data was not included even though p63 motif is a statistically significant predictor (P value <0.0001). Indeed on its own the sequence information accounts for 14% of the variability in p63 binding (Additional file 5: Table S2, Additional file 6: Table S3). We therefore performed additional analysis to ascertain the importance of p63′s motif in dictating DNA binding, when taking into account epigenetic information. We divided all accessible genomic locations as determined by DNase-Seq into three groups based on the presence of a strong, weak, or the absence of a p63 motif. Our results indicate that the presence of a strong or weak p63 motif leads to stronger p63 occupancy (Figure 5A). This finding that p63 DNA-sequence motif is an important determinant of *in-vivo* binding is difficult to reconcile with the fact that p63 motif was not considered a strong predictor in our statistical models. One possible explanation for this discrepancy is that the chromatin datasets we use in our models have already embedded in it the DNA sequence motif information. Indeed, using our regression model, we predicted p63 occupancy for each group and showed that predicted p63 occupancy is also higher for sites containing a strong or weak p63 motif (Figure 5B). This provides further support for the notion that H3K4me1, DNase and DF variables in our statistical models already account for the information represented by the p63 consensus motif. Thus accessible sites with a strong or weak motif are more accessible and have higher deposition of H3K4me1 than sites lacking a p63 motif (Figure 5C, 5D). Taken together, these results lead us to postulate that p63 might exist as part of a chromatin remodeling complex, which creates a distinct epigenetic architecture at its binding sites (see Discussion for details).

### Identification of p63 Cooperating TFs

It is well established that many TFs often act in a combinatorial fashion to govern tissue-specific gene expression. Hence, we wanted to examine the repertoire of p63 associated TFs that might play such role in modulating p63 binding and possibly influencing p63-dependent gene expression in keratinocytes. We therefore used a combination of in-silico analysis of the p63 binding sites and careful data mining of large-scale genomic datasets such as RNA-Seq and ChIP-Seq from the ENCODE project. We posit that the TF that are likely to directly cooperate with p63 will have the following features. First, their DNA binding motifs will likely be enriched at p63 bound elements, keeping in mind that co-occurrence of such motifs adjacent to p63 sites is not a prerequisite for such interactions. Second, their in-vivo binding profiles will overlap with p63 binding profile. Here we reasoned that TF binding to some extent could be extrapolated from ChIP-Seq in other cell types, as it has been shown that TFs share a large number of common binding sites across different cell lines [46]. Finally, we contend that the relevant TF should be expressed in keratinocytes.

**Figure 6:**
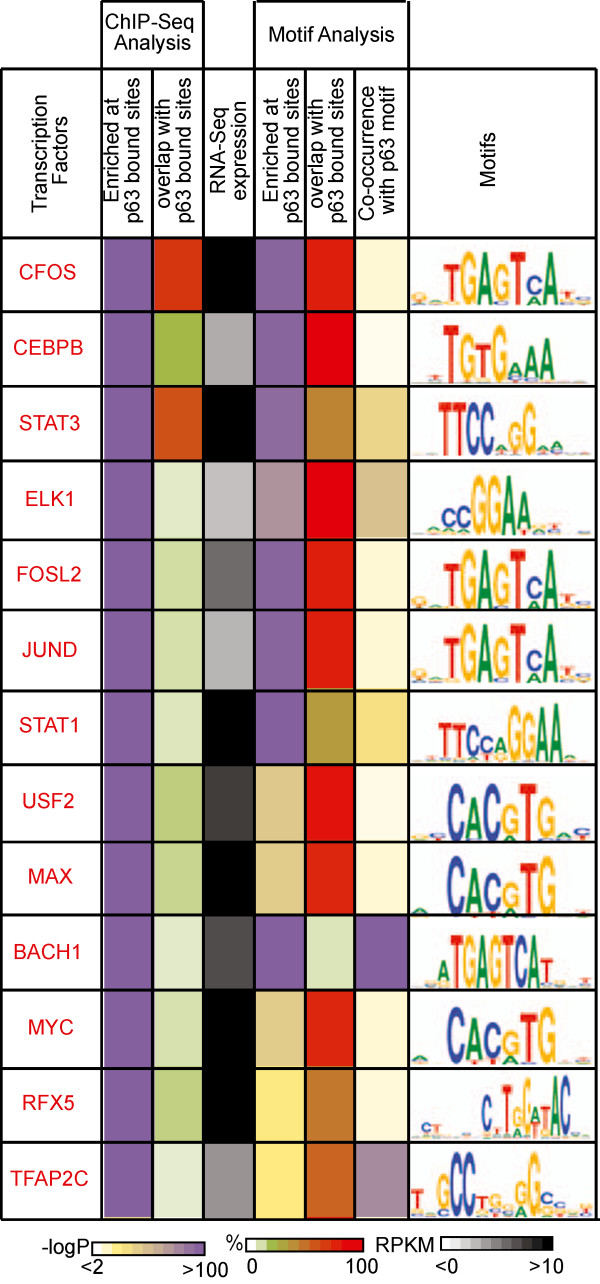
**Cooperating TFs of p63.** 13 TFs corresponding to 10 motifs (CEBPB, AP1(CFOS, FOSL2,JUND), BACH1, AP2(TFAP2C), STAT1, STAT3, MYCMAX (MAX, c-MYC), USF(USF2), RFX(RFX5), ELK1) were determined as potential cooperating TFs for p63. The characteristics that determine these TFs as potential cooperating TFs are: 1) enrichment of TF binding at p63 targets (P value <0.01 & overlap >5%) - as measured by ChIP-Seq from any available cell-line, 2) high expression in keratinocytes (RPKM >2) - as determined by RNA-Seq in NHEK cell-line, 3) enrichment of the TF’s motif at p63 targets (P value <0.01 & overlap >5%) and co-occurrence with p63 motif (P value <0.01). The TFs are sorted in descending order by an average percentage rank calculated across the different analysis.

Applying this criterion, we examined 631 TFs for which ChIP and/or expression data was available from the ENCODE project. Of these, 467 were enriched at p63 targets as determined by their in-vivo binding profile or *in silico* motif analysis. However, we rejected 404 of these TFs as potential cooperating TFs because they were either not expressed in keratinocytes (RPKM <2) or not enriched by both ChIP-Seq and motif analysis (P value >0.01 or overlap <5%). An additional 50 TFs were also not considered from further evaluation due to ill-defined/unknown DNA binding motifs or missing ChIP-Seq data. This shortlisted the potential p63 cooperating TFs to 13 TFs, which had both an enriched motif and in-vivo binding signal (P value <0.01 and overlap >5%) at p63 bound locations in NHEK cells (Additional file 7: Table S4). These 13 TFs include: CEBPB (C/EBP family); CFOS, FOSL2, and JUND (AP1 family); BACH1 (bZIP family); TFAP2C (AP2 family); STAT1 and STAT3 (STAT family); MAX, c-MYC, and USF2 (bHLH family), RFX5 (RFX family); ELK1 (Ets family) (Figure 6). In this context, it is important to note that several of the predicted TF discovered by our analysis, such as AP1, AP2, MYC, STAT and C/EBP have been previously linked to p63 and keratinocyte biology [9, 18, 47–50]. On the other hand, some of the other potential p63 cooperating TFs such as RFX5, ELK1 and BACH1 are new members of this class. They represent an interesting group for which much less is known about their expression and function in keratinocytes and any possible correlation with p63. One particularly intriguing p63 cooperating TF is RFX5, which is highly expressed in NHEK cells as evident by the RNA-Seq data (Figure 6). Furthermore, a recent study has also shown that RFX5 mRNAs and protein are preferentially expressed in the p63-rich basal cells of the human epidermis further raising the prospects of a functional interplay between these two TFs [51].

### The potential role of p63 in modulating different biological functions in coordination with various cooperating TFs

**Figure 7:**
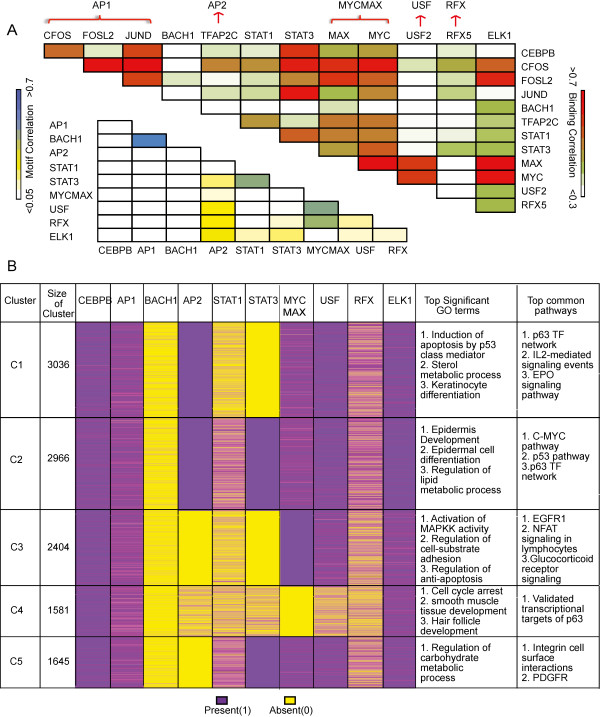
**p63 functions with multiple cooperating TFs. (A)** A correlation matrix for the 13 probable p63 cooperating TFs at the 11,632 p63 locations. *Upper triangle* shows Pearson’s correlation coefficient calculated between ChIP-Seq tag densities for each pair of TFs. For BACH1 and FOSL2, ChIP-Seq signal are from K562 and A549 cell-lines respectively. While for the rest of the 11 factors, ChIP-Seq signal is from Hela-S3 cell-line. *Lower triangle* shows correlation between motif scores generated by Patser. **(B)** The 11632 p63 bound locations are clustered by the presence (purple) or absence (yellow) of the 10 cooperating TFs motifs (CEBPB, AP1, BACH1, AP2, STAT1, STAT3, MYCMAX, USF, RFX, ELK1), in a 1 kb window centered at p63 binding site.

p63 is involved in myriad biological functions and it is possible that this diversity of p63 function is brought about by in part by different subtypes of p63-TF complexes. We therefore constructed a correlation matrix of the 13 TFs based on their *in-vivo* binding signal and their DNA-binding motif scores at the 11,632 p63 bound locations (see Methods for details). The 13 TFs correspond to 10 unique DNA-binding motifs (CEBPB, AP1, BACH1, AP2, STAT1, STAT3, MYCMAX, USF2, RFX and ELK1). There appears to be multiple complexes with unclear distinctions between them (Figure 7A). Two distinct complexes containing overlapping TFs could be identified. The first group comprised of C/EBPB, JUND, CFOS, and STAT3, whereas the second complex was primarily represented by ELK1, FOSL2, and MYC-MAX. We next asked if there were different sub-groups of genomic targets where p63 was bound in coordination with these distinct cooperating TFs. Upon clustering the 11632 p63 targets, 5 clear groups emerged (C1 to C5) based on the presence/absence of the cooperating TF motifs (Figure 7B, Additional file 3: Table S1). AP2, STAT3 and MYCMAX seem to be the driving force for the clustering, whereas CEBPB, AP1 and ELK1 motifs were quite ubiquitous in their presence. Further annotating the clusters using GREAT allowed us to associate specific groups of p63 targets with distinct biological functions, based upon Top GO terms and significant pathways. The complete annotation for each group of p63 targets and the type of complex it represents is shown in Figure 7B.Our clustering analysis revealed an interesting and complex correlation between p63, its network of associated cooperating TFs and the nature and physiological role of the p63 target genes. Case in example is AP2. It seems to be an important co-player of p63 in regulating genes involved in keratinocyte differentiation and epidermis development (clusters C1 and C2), modestly linked with hair follicle development and cell cycle arrest (C4) but not at all involved in the activation of MAPKK activity (C3) or regulation of carbohydrate metabolic process (C5). Similarly, while STAT1 and STAT3 seem to share similar co-regulatory function as evident by their enrichment in clusters C2 and C5, there are also clear differences between these two closely related family members based on their enrichment patterns for clusters C1, C3 and C4. This point is further illustrated by the correlation matrices made in Figure 7A. In contrast, binding motifs for C/EBP, ELK1 and AP1 did not seem to exhibit a preference for any of the particular clusters representing different GO terms or biological pathways. Interestingly, none of the 5 clusters showed any difference in terms of p63 motif strength or the type of regulatory regions (data not shown).

**Figure 8:**
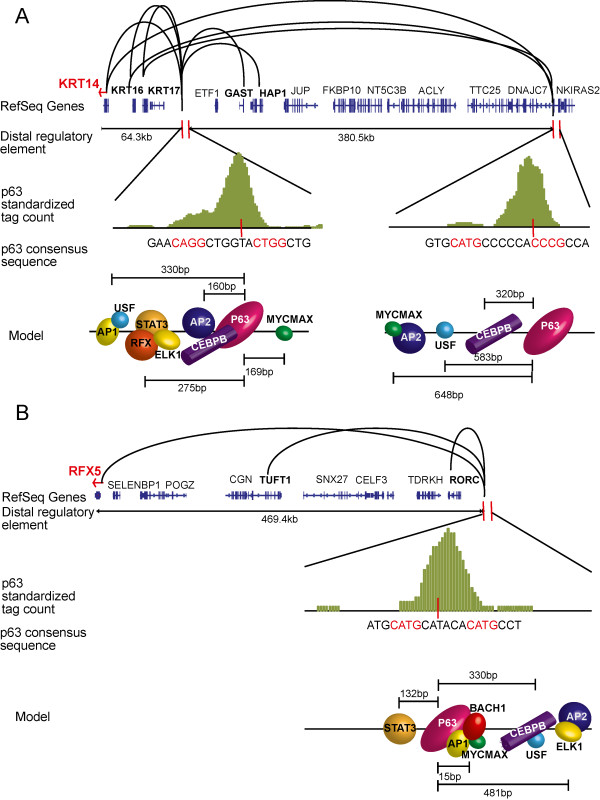
**Model of p63 binding at distal regulatory elements of keratin-14 and RFX5.** *Histograms* in green show p63 tag density in a 1 kb window at the regulatory region. Loops links the distal regulatory regions to their target promoter(s) as mapped by the ENCODE project [53]. **(A)** 2 p63 binding sites 64.3 kb and 380.5 kb upstream are linked to keratin-14 (KRT14). These regions also regulate other genes, among them keratin-16 (KRT16) and keratin-17 (KRT17). Motif analysis reveals other TFs binding within the 1 kb window. **(B)** p63 binds 469.4 kb upstream to RFX5 at a distal regulatory element linked to RFX5 promoter. This region is also linked to TUFT1 and RORC promoters. Motif analysis shows p63 binding in complex with AP1, E-box binding factors and BACH1 on a weak canonical motif. Additional motifs are also found within the 1 kb window.

One obvious limitation of our approach is the fact that for the purpose of the aforementioned studies, a specific p63-binding site was assigned to be regulatory element for the nearest gene, often without any supporting experimental data. However it has been shown, that linking distal enhancer elements to the nearest gene, as is the norm, might not always be an accurate representation of the regulatory landscape [52]. There is an absence of chromatin interactions data such as those obtained from chromosome conformation capture (3C), in keratinocytes. We therefore virtually linked the distal regulatory elements to their target genes, by exploiting the characteristic that regulatory regions become DNase hypersensitive (DHS) in synchrony with their target promoters. To examine this further, we first retrieved the global map of distal DHS-to-promoter connections as generated by the ENCODE consortium [53]. Overlapping this map with the p63 ChIP-Seq data allowed us to link 4011 p63 bound distal genomic regions to their putative target promoters/genes (Additional file 8: Table S5). Further analysis revealed interesting facets of long-range interactions between distal p63-bound elements and the promoter as evident by the *KRT14* gene, a known p63 target [14, 17]. While prior studies have focused primarily on the proximal regions 5′ of the *KRT14* gene, including a well-characterized p63-bound enhancer ~1.4 kb upstream [14, 17], we discovered 7 novel distal p63-bound elements that are located quite far away (~60 kb to ~450 kb) as predicted by the DHS-to-promoter connections (Additional file 8: Table S5). Two of these p63-bound genomic segments, which are predicted to be enhancers for *KRT14* are shown in Figure 8A. These regulatory regions also contained potential binding sites for some of the p63 cooperating TFs such as CEBPB, c-MYC and AP2. Interestingly these two distal p63-bound elements are not exclusive to the *KRT14* gene since they are also putatively linked to related *KRT16/ KRT17* genes, which are located in a relatively closely spaced genomic cluster (Figure 8A). This raises an interesting possibility that a specific p63-bound regulatory region (an enhancer for e.g.) might be commonly utilized to coordinate the regulation of multiple closely related genes in keratinocytes.

We also discovered possible new target genes under the control of p63-bound distal regulatory regions that otherwise would have been missed by the conventional strategy of assigning a p63-regulated element to its closest gene. As shown in Figure 8B, one such interesting candidate is RFX5, a novel cooperating TF of p63 as discussed above. p63 binds a regulatory element 469.4 kb upstream of the *RFX5* gene and motif analysis suggests that AP1, E-box binding factors and BACH1 are part of the transcriptional complex at this site (Figure 8B). Hence, there might exist a transcriptional regulatory loop whereby p63 in cooperation with additional TFs activates RFX5, which then in turn, modulates p63 binding to its target sites. Similar to the case with the *KRT14* gene, this distal regulatory element for RFX5 is predicted to be also linked to the *TUFT1* and *RORC* genes. In the future in-depth studies such as 3C experiments will help to confirm these distal enhancer-promoter interactions and to firmly establish the true identity of the p63-driven gene network in keratinocytes.

## Discussion

This study aimed to decipher the mechanics of p63 binding by determining the minimal *in-vivo* motif required for binding, distinguishing between chromatin architecture of bound and unbound motifs and identifying cooperating TFs that modulate p63 biological activity.

### p63 requires a full site for binding to target sites

The transcription factor p63 binds as a homotetramer to two decamers RRRCRWGYYY, RRRCWYGYYY separated by a 0–15 base pair spacer region. We found that the p63 sites containing the decamer pair with intervening spacers was much less prevalent (16.4%) than those where the two half sites were juxtaposed to each other (73.3%). A very small subset (only 8 genomic locations) of p63 binding sites consisted of only a half site (1 decamer) (Additional file 2: Figure S2). This is in contrast to a similar study that found 3-4% of p53 binding sites having a half site [54]. This observation can be explained by a slight difference in the consensus motifs of the two factors, which also results in 3 fold lower binding affinity of p63, in comparison to p53 [55]. One possibility is that the dimer-dimer interactions are important for p63 DNA binding specificity and therefore p63 requires a full site to bind DNA efficiently. Indeed, such differences in the DNA-protein interactions among p53 family members are quite evident from recent structural studies with p73 [56]. Interestingly, not only the distance between two p73 half-sites influences the p73 quaternary structure, but tellingly transcriptional activity is also more affected by spacer length in p73 response element than in p53. Finally, it is worth noting that ~10% of p63 ChIPed sites in keratinocytes do not have a recognizable p63 binding sequence suggesting that the p63 binding at these sites is driven by indirect mechanisms that might involve other DNA-binding TFs and/or non-canonical p63 motifs.

### The p63 consensus motif is not required for predicting binding events in keratinocytes

We utilized two computational modeling approaches to uncover key characteristics defining p63 binding sites. First, we utilized discriminant modeling that allowed us to predict p63 binding as a binary score (presence or absence). Second, in a parallel strategy, we used regression modeling that predicted the degree of p63 occupancy. Our approach was distinct from other published methods in that we trained our statistical models on random genomic sites which might or might not have p63 binding sequences [57]. This allowed our models to include the 10% binding sites that do not have p63 DNA-binding motif. Surprisingly, we found that our final models constituting only chromatin marks (H3K4me1, H3K27me3 and accessibility data) predicted p63 binding with high accuracy (Discriminant Model - 89.8%(±0.3) sensitivity, Regression model – 0.083(±0.001) MSE). Adding sequence information to the models, did not lead to any significant improvement even though p63 motif is a statistically significant predictor (Additional file 5: Table S2, Additional file 6: Table S3). One possible explanation is that in keratinocytes, regions of the genome that have a functional p63 motif are on average more accessible and marked with active chromatin marks. This result is not surprising if p63 is a key component of the regulatory complex that is involved in remodeling the chromatin at its binding sites. Indeed p63 target sequences dictate higher nucleosome occupancy than random genomic sequences according to the nucleosome-DNA interaction model (Figure 4). It can be then hypothesized that p63 binding shifts the nucleosomes creating an accessible active chromatin structure at its targets. Support for such a role for p63 comes from a recent study, which examined the selective loss versus gain of DHSs targeted by lineage regulating TFs during lineage differentiation from ESCs [58]. While the recognition landscape for p63 remains largely unchanged during development of all other lineages, there is a significant and selective gain of p63 binding elements in the DHSs of the human skin keratinocytes, which represent the ectodermal lineage. Such deterministic function of p63 is further evident by data showing that p63 in combination with KLF4 can efficiently convert human fibroblast into keratinocytes [59]. These interesting correlative findings, together with our results presented here strongly suggest that p63 functions as part of a pioneering complex which can target and remodel chromatin at many of its sites.

### Binding of p63 in coordination with cooperating TFs

It is likely that the p63-depedent regulation of target genes in keratinocytes requires co-operation of other TFs. We have used a multi-pronged approach to identify such p63-associated cooperating TFs by processing data from NHEK RNA-seq, available ENCODE ChIP-seq and computational prediction methods based on TF motifs. Our analysis led to a few surprising observations about the identity of candidate TFs that were likely to be involved in p63-genomic interactions. One striking result from our study is that many of the p63-associated factors belong to broadly expressed family of TFs such as AP1, AP2, MYC and STAT rather than highly tissue-specific factors. Although at first glance, this result may seem disappointing, we think that given the master regulatory role of p63, such a finding makes biological sense. Indeed, given the fact that p63 is highly expressed in a lineage-restricted fashion and plays a crucial role in dictating keratinocyte cell fate, it is conceivable that some of the p63-associated cooperating TFs might just provide ancillary role in regulating gene expression. Another interesting possibility is that the keratinocyte-specific gene expression is mediated by a combinatorial interaction of multiple TFs as suggested by prior studies [60]. However it is important to stress that many of the broadly expressed TFs such as AP1 and AP2 do have keratinocyte-specific roles that are often masked due to functional redundancy from expression of multiple family members [47, 49]. Future functional studies on these TFs that are part of the p63-driven transcriptional network, including ones that are relatively under studied such as RFX5 will shed important insights into gene regulatory mechanisms in keratinocytes.

## Conclusion

Despite the wealth of information obtained from our data-mining studies, long term follow-up experimental studies are needed to better elucidate the p63 TF network and the role of chromatin in regulating myriad biological functions of p63. Unraveling the complex nature of the distal regulatory elements such as enhancers, which are by far the most common sites of p63 binding is a formidable challenge. The new insight into the dynamic interplay between p63, its many cooperating TFs and the local chromatin environment, as reported here is the first step towards tackling such challenges.

## Methods

### Determining p63 binding profile in the genome

Global p63 binding locations in keratinocytes was determined from ChIP-Seq datasets generated by Kouwenhoven et al. [16]. The Illumina FASTQ sequencing files from three independent replicates were aligned to hg19 with bowtie [30] with the following parameters: m = 1 (i.e. removes all those alignments with more than one match). P63 binding locations were then identified in each experiment under stringent conditions with MACS (cutoff p-value = 1e-10) [61]. The 11632 locations that were common in all three replicates were used in this study.

### Finding p63 motif in p63 bound locations

Patser [62] was used to search for the occurrence of p63 canonical motif (defined as 2 decamers (RRRCRWGYYY, RRRCWYGYYY) with zero spacer in between) [20] in a 500 bp window around the 11632 p63 bound locations. To determine a cutoff score, above which the motif would be termed as a significant match, we created a background model with 100000 random genomic sites. The motif score for which the probability of any stronger motif occurring by random chance would be less than 0.01, was selected as the cutoff for strong motifs (For p63 - cutoff score of 7.14). A relaxed cutoff of 2.24, corresponding to a weaker motif (0.1 probability of occurring by random chance) was also determined. We then repeated the above procedure for possible non-canonical p63 motifs. For this we modified the position weight matrix (PWM) used earlier by inserting spacers (1–15 with 0 weight for each base, i.e. each base assumed to be equally likely) and again used the background model to calculate cutoffs and determine significant matches. We also did this for a half-site (only 1 decamer).

### Identifying the chromatin profile at p63 targets

Histone modification ChIP-Seq data for 5 active histone modifications (H3K4me1, H3K4me2, H3K4me3, H3K9ac, H3K27ac) and 2 repressive histone modifications (H3K9me3, H3K27me3) in NHEK (Normal Human Epidermal Keratinocytes) cell-line were obtained from ENCODE [31]. The coordinates for the 11632 p63 bound locations and 30,000 negative genomic sites (any genomic site not within 5 kb of a p63 bound location was termed as a negative site) that had strong p63 canonical motif were obtained. The histone marks were plotted for a 1 kb window at 10 bp resolution in standardized tag count space.

### Clustering p63 targets by histone signature

An average signal across a 1 kb window centered at the p63 binding site was plotted for the 5 active (H3K4me1, H3K4me2, H3K4me3, H3K9ac, H3K27ac) and 2 repressive (H3K9me3, H3K27me3) histone modifications. Using k-means clustering algorithm, implemented in Cluster 3.0 software package [63], with k = 2, the p63 targets were divided into Group A which contained high overall signal for the different histone modifications and Group B, which contained sites with lower overall signal. For each of the two groups, the seven histone modifications were standardized and again plotted in count space, this time at 10 bp resolution. This was done to take into account the spatial arrangement of the histone modifications. Both the groups were individually clustered using k-means algorithm, with k = 4. Pearson correlation was calculated between each pair of clusters, in both directions, for both the groups. Clusters with Pearson correlation higher than 0.9 were grouped together.

### Training and test dataset for computational models of p63 binding

The 100,000 random genomic locations were filtered to generate 94760 negative sites (Sites not within 5 kb of a p63 bound location were termed as negative sites). These, along with 11,632 p63 bound locations were used to train and test our Fisher’s discriminant and regression models. Five active (H3K4me1, H3K4me2, H3K4me3, H3K9ac, H3K27ac) and two repressive (H3K9me3, H3K27me3) histone modifications as measured by ChIP-Seq, along with accessibility as measured by DNase-Seq and FAIRE-Seq, in NHEK cell-line were plotted as an average signal across a 1 kb window for each of the 106,392 genomic coordinates, in sqroot space. All the datasets were standardized to 30 million tag count so as to be comparable to each other. These 9 datasets along with interaction terms (calculated as product of two datasets for all possible combinations – e.g. DF (DNase*FAIRE)) were used as predictor variables along with the p63 canonical motif score as generated by Patser, for the computational models. p63 tag count in standardized sqroot space was the response variable for the regression model and a categorical variable with two possible values (0 (bound) and 1 (unbound)) was the response variable for the discriminant model. The 94,760 negative genomic coordinates and 11,632 positive genomic coordinates were then randomly divided into training and test datasets, such that the training dataset had 45,000 negative genomic sites and 5000 positive genomic sites. For the discriminant model, the training dataset was further filtered so as to keep only those negative genomic locations that had p63 tag count (standardized sqroot space) less than 0.25, resulting in 30000 negative genomic sites and 5000 positive genomic sites. This random division into training and test datasets was then repeated 10 times to obtain the mean sensitivity (true positive rate) and mean specificity (true negative rate) for the discriminant model and mean MSE (mean square error) and mean R^2^ (fraction of variance in p63 occupancy explained by the model) for the regression model.

### Fisher’s discriminant model

DISCRIM procedure in SAS statistical software package was used to construct a Fisher’s Discriminant Model. Using all the variables in the discriminant training dataset (9 chromatin features (H3K4me1, H3K4me2, H3K4me3, H3K9ac, H3K27ac, H3K9me3, H3K27me3, DNase, FAIRE) and one interaction term (DNase*FAIRE)), we created a full discriminant model. To simplify the model, we used the STEPDISC procedure to determine 8 variables with significant predictive power. They were used to make the significant chromatin marks model. This was further simplified into the Best 3 variable model, by using only the top 3 variables (H3K4me1, DNase, H3K27me3), with the highest predictive power. The Best 3 variable model was then tested on the test dataset.

### Regression model

REG procedure in SAS statistical software package was used to create a regression model for p63 occupancy based on the chromatin features (no sequence information). We started with a model with no predictors and used stepwise selection to add significant predictors to the model, starting with the one that had the smallest P value (till P value for entry was less than 0.01). At each step the P value for exit was also calculated and the predictor was retained only if its P value was less than 0.01. The 9 chromatin features (H3K4me1, H3K4me2, H3K4me3, H3K9ac, H3K27ac, H3K9me3, H3K27me3, DNase, FAIRE) and one interaction term (DNase*FAIRE) were all found to be statistically significant and formed the full regression model. To simplify this model and find the features with the most predictive power we then used MAXR (Maximum R^2^ Improvement) selection method, with parameter STOP = 3, to determine the best 3 variable model for predicting p63 occupancy.

### Identifying p63 cooperating TFs

We utilized the motifs database by Genomatix software and used Patser to search for the occurrence of 900 TF motifs in a 1 kb window centered at the 11632 p63 bound locations (test set) and 94760 negative genomic sites (background set). Chi-Square test was done to statistically determine which of the TFs motifs were enriched at the p63 binding sites versus the background. % overlaps of the motifs with p63 binding profile were also determined. We then examined for co-occurrence of the TFs motifs with p63 motif. For this our test set contained 8,375 p63 bound locations that had at least a weak p63 motif (score greater than 2.24) and the background set contained 515,933 unbound genomic sites (containing a weak motif). Again chi-square test was done to find the statistically enriched motifs within 100 bp of p63.

The second step to our approach was to use the *in vivo* binding profiles of TFs, to find the potential cooperating TFs of p63. 264 ChIP-Seq experiments as carried out by ENCODE, capturing an in-vivo binding profile of TFs across 30 cell-lines were used for this analysis. Overlaps were determined between these experiments and p63 binding profile. The same was repeated for the 94760 negative genomic sites. Chi-square test determined the TFs showing enriched binding at p63 targets. We also used NHEK RNA-Seq data to eliminate TFs with RPKM <2 in keratinocytes.

### Correlation matrix of cooperating TFs

*ChIP-Seq correlation matrix:* 13 TFs (CEBPB, CFOS, FOSL2, JUND, BACH1, TFAP2C, STAT1, STAT3, MAX, c-MYC, USF2, RFX5, ELK1) were identified as the most probable cooperating TFs of p63. ChIP-Seq alignment files in Hela-S3 cell-line were obtained from ENCODE via the UCSC genome browser for 11 of the 13 TFs. For BACH1 and FOSL2 we used the alignment files from K562 and A549 cell-lines respectively. An average signal across a 1 kb window was plotted for each of the 13 factors across the 11632 p63 bound locations. Then Pearson correlation coefficient (r) was calculated for each pair. *Motif correlation matrix:* The 13 TFs corresponded to 10 Position weight matrices (PWMs) (CEBPB, AP1 (CFOS, FOSL2, JUND), BACH1, AP2 (TFAP2C), STAT1, STAT3, MYCMAX (MAX, c-MYC), USF(USF2), RFX(RFX5), ELK1). For each PWM, a Patser generated motif score was obtained for the 11,632 locations. Again, Pearson correlation coefficient (r) was calculated for each pair.

### Clustering p63 targets by cooperating TFs motifs

The 10 PWMs (CEBPB, AP1, BACH1, AP2, STAT1, STAT3, MYCMAX, USF, RFX, ELK1) were used to search for the occurrence of REs in a 1 kb window centered at each of the 11632 p63 binding sites. The default cutoffs determined by Patser based on the information content of each of the weight matrices were used to assign a binary score of 0 (Motif absent) and 1 (Motif present) across the 11632 genomic locations. This binary matrix was then clustered using k-means algorithm, implemented in Cluster 3.0 software package [63], with k = 5.

## Electronic supplementary material

Additional file 1: Figure S1: Background Model for determining strong p63 motif. Patser is used to search for the occurrence of p63 canonical motif in a 500 bp window around the 11632 p63 bound locations and 100000 random genomic sites. A relative frequency chart of the motif scores is plotted for both the bound and background locations. Strong motifs are defined as occurring in less the 1% of random background sequences while weak motifs occur in less than 10% of background sequences. (PDF 132 KB)

Additional file 2: Figure S2: P63 binds to the canonical p63 motif without a spacer. Patser is used to search for the occurrence of p63 non-canonical full motifs (2 decamers with a spacer sequence of length 1–15 nucleotides) and half site (only 1 decamer). (**A)** Frequency of p63 binding sites containing zero spacer, i.e. canonical motif (blue), 1–15 spacer (red) and half-site (green). (**B)** P63 binding sites divided by type of motif. 73.3% have canonical motif, 16.36% have non-canonical motif, only 0.07% have half site and the rest do not have a p63 motif. (PDF 832 KB)

Additional file 3: Table S1: P63 Master Annotation Dataset. Columns A-C contain the genomic coordinates (hg19) of the 11632 p63 binding sites. These regulatory regions are assigned a p63 motif score based on Patser (column D). They are linked to the nearest gene (column E, column F) and annotated using chromatin based segmentation (column G) [39]. Each region is assigned to a histone cluster (column H) as shown in Figure 2 and to a cofactor cluster as depicted in Figure 7B (column I). Presence (1) / absence (0) of individual cofactors motifs at each regulatory region is then shown (column J-S). (CSV 962 KB)

Additional file 4: Figure S3: Chromatin profiles are associated with gene expression. p63 bound locations are clustered by 5 active (H3K4me1, H3K4me2, H3K4me3, H3K9ac, H3K27ac) and 2 repressive (H3K9me3, H3K27me3) histone modifications (Figure 2). The 5 groups (A1, A2, B1, B2, B3) of p63 targets are annotated to the nearest genes by GREAT. Box plots of gene expression as determined by RNA-Seq in keratinocytes, is shown. (PDF 82 KB)

Additional file 5: Table S2: Discriminant Models. Fisher’s Discriminant models are made in SAS statistical software using DISCRIM procedure with 11 predictors. The *full models* use all the 10 chromatin marks. *Significant chromatin marks models* use 8 chromatin marks (Significant, if P value <0.0001). *Regression 3 Model* is made with the three marks (H3K4me1, DNase, DF) used to make the final regression model. *Best 3* is the final discriminant model used to predict p63 binding, made with the three predictors having the highest predictive power (H3K4me1, DF, H3K27me3) according to STEPDISC procedure in SAS statistical software. Sensitivity and Specificity are used to judge the accuracy of the models. (PDF 411 KB)

Additional file 6: Table S3: Regression Models. Regression models are made in SAS statistical software using REG procedure. The *full models* use all the 10 chromatin marks as predictors (Each predictor is significant at P-value 0.0001). *Best 3 Model* is made with the three marks (H3K4me1, DNase, DF) that together have the highest predictive power (Accounts for 74.8% variability in p63 binding, MSE = 0.08). Finally models with each individual predictor are also made. (PDF 885 KB)

Additional file 7: Table S4: p63 Cooperating TFs Dataset. The 467 TFs enriched by either ChIP-Seq analysis or motif analysis (P value <0.01) are divided into 4 groups - **Group A**: 13 TFs that are a) expressed in keratinocytes (RPKM >2), b) their in-vivo binding profiles are enriched (P value <0.01 & overlap >5%), c) motifs are enriched at p63 targets (P value <0.01 & overlap >5%) and co-occurs with p63 motif (P value <0.01). **Group B** (Incomplete Analysis): 11 TFs for which DNA binding motifs are unknown, **Group C** (Incomplete Analysis): 39 TFs for which there is no ChIP-Seq data and **Group Rejected**: 404 TFs rejected because a) they are not expressed in keratinocytes (RPKM <2) or b) they are not enriched by ChIP-Seq or motif analysis (P value >0.01 or overlap <5%). In the table, columns A-C mention TF name, -logPvalue of enrichment and % overlap of the in-vivo binding profile of the TF with p63 binding sites. Column D is the expression of the factor in keratinocytes. Columns E-F contain -logPvalue of enrichment and % overlap of the TF motif with p63 bound sites. Column G is the -logPvalue of enrichment of the co-occurrence of TF motif with p63 motif (i.e. within 100 bp). The motif name is in column H. Any missing data is represented by -1. The TFs are arranged in descending order by an average percentage rank calculated across columns B-G for each of the 4 groups. (XLS 108 KB)

Additional file 8: Table S5: Distal p63 binding sites linked to target promoters dataset. This .csv file has 9106 lines of data (excluding header), for 4011 p63 binding sites (distal regulatory regions), that are linked to at least one target promoter in accordance with the global map of distal DHS-to-promoter connections generated by the ENCODE consortium [53]. Columns A-C contain the genomic coordinates of each of the 4011 p63 binding sites (hg19). Columns D-F represent the genomic coordinates of the linked promoter. Gene names are given in column G. The distal regulatory regions might be linked to more than one promoter/gene and are therefore represented as multiple records in the dataset, with different promoter coordinates for each repetition. (CSV 497 KB)
