# Supplementary figures and images for: Role of chromatin and transcriptional co-regulators in mediating p63-genome interactions in keratinocytes
Source: BMC Genomics. 2014 Nov 29;15(1):1042. doi: 10.1186/1471-2164-15-1042 (PMC4302094; doi:10.1186/1471-2164-15-1042)

Frequency Distribution curve of p63 motif scores

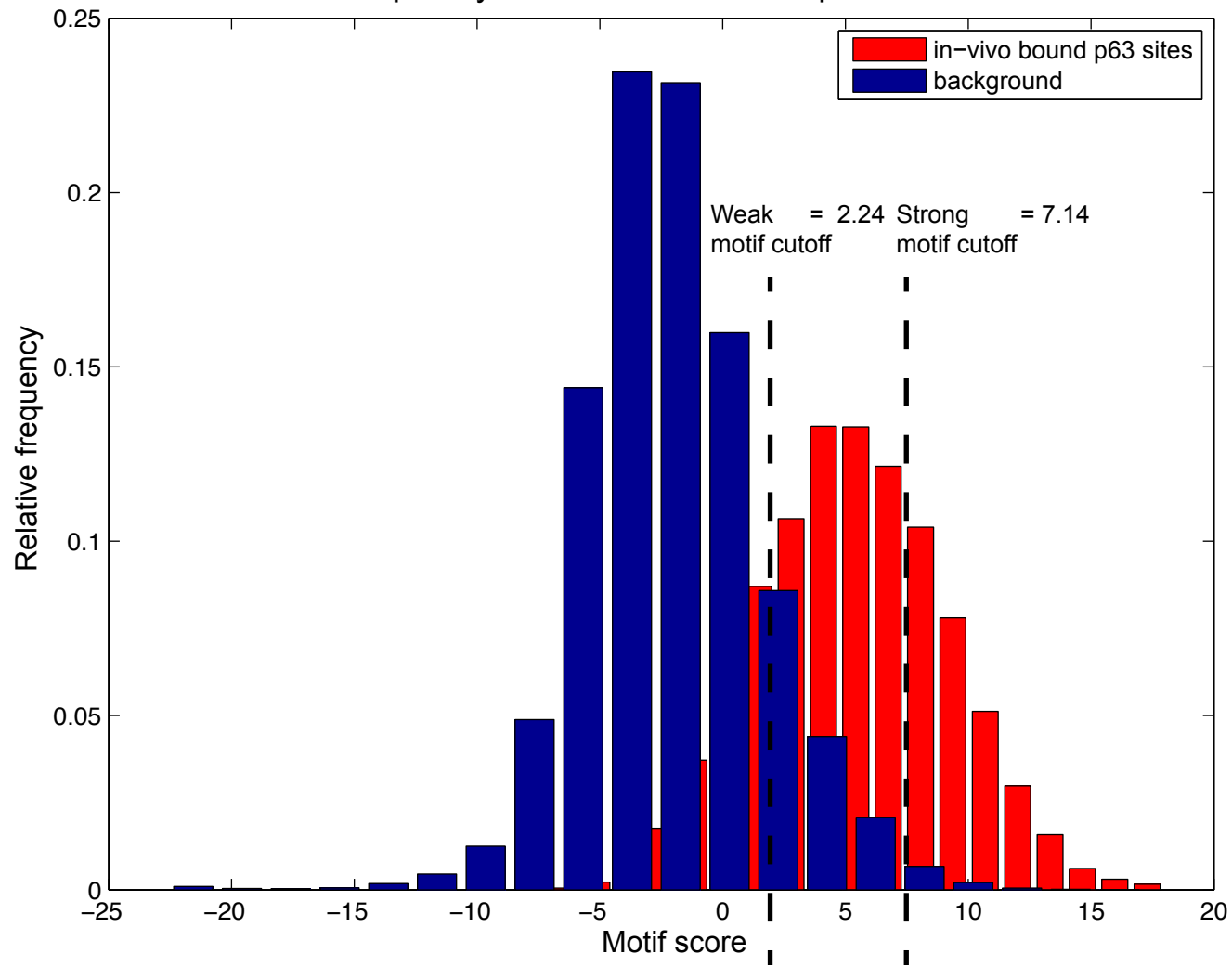

Supplement: Supplementary file 1 — Additional file 1: Figure S1: Background Model for determining strong p63 motif. Patser is used to search for the occurrence of p63 canonical motif in a 500 bp window around the 11632 p63 bound locations and 100000 random genomic sites. A relative frequency chart of the motif scores is plotted for both the bound and background locations. Strong motifs are defined as occurring in less the 1% of random background sequences while weak motifs occur in less than 10% of background sequences. (PDF 132 KB) [file 12864_2014_6850_MOESM1_ESM.pdf]

A

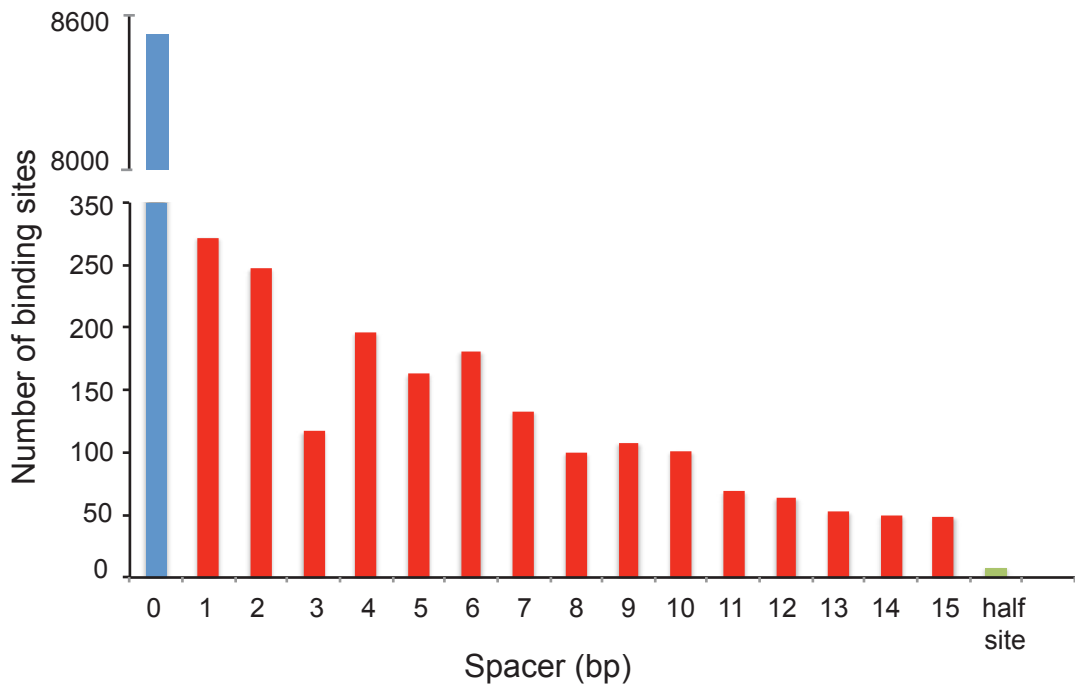

B

P63 binding sites (11632)

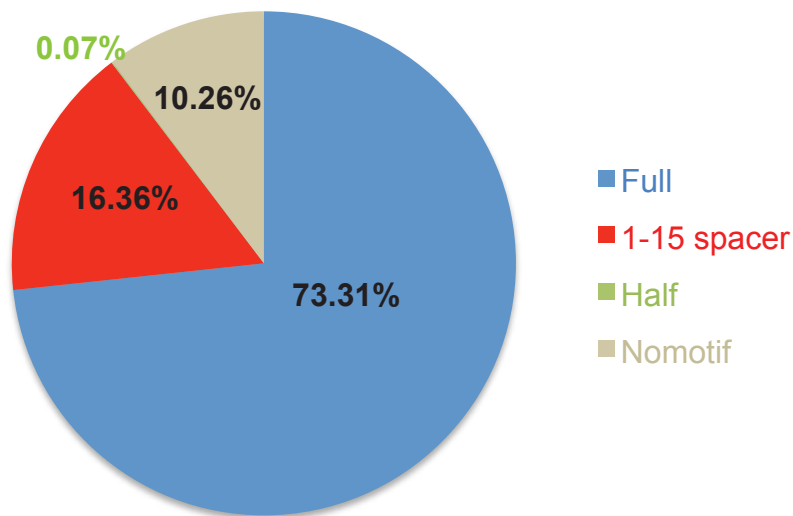

Supplement: Supplementary file 2 — Additional file 2: Figure S2: P63 binds to the canonical p63 motif without a spacer. Patser is used to search for the occurrence of p63 non-canonical full motifs (2 decamers with a spacer sequence of length 1–15 nucleotides) and half site (only 1 decamer). (A) Frequency of p63 binding sites containing zero spacer, i.e. canonical motif (blue), 1–15 spacer (red) and half-site (green). (B) P63 binding sites divided by type of motif. 73.3% have canonical motif, 16.36% have non-canonical motif, only 0.07% have half site and the rest do not have a p63 motif. (PDF 832 KB) [file 12864_2014_6850_MOESM2_ESM.pdf]

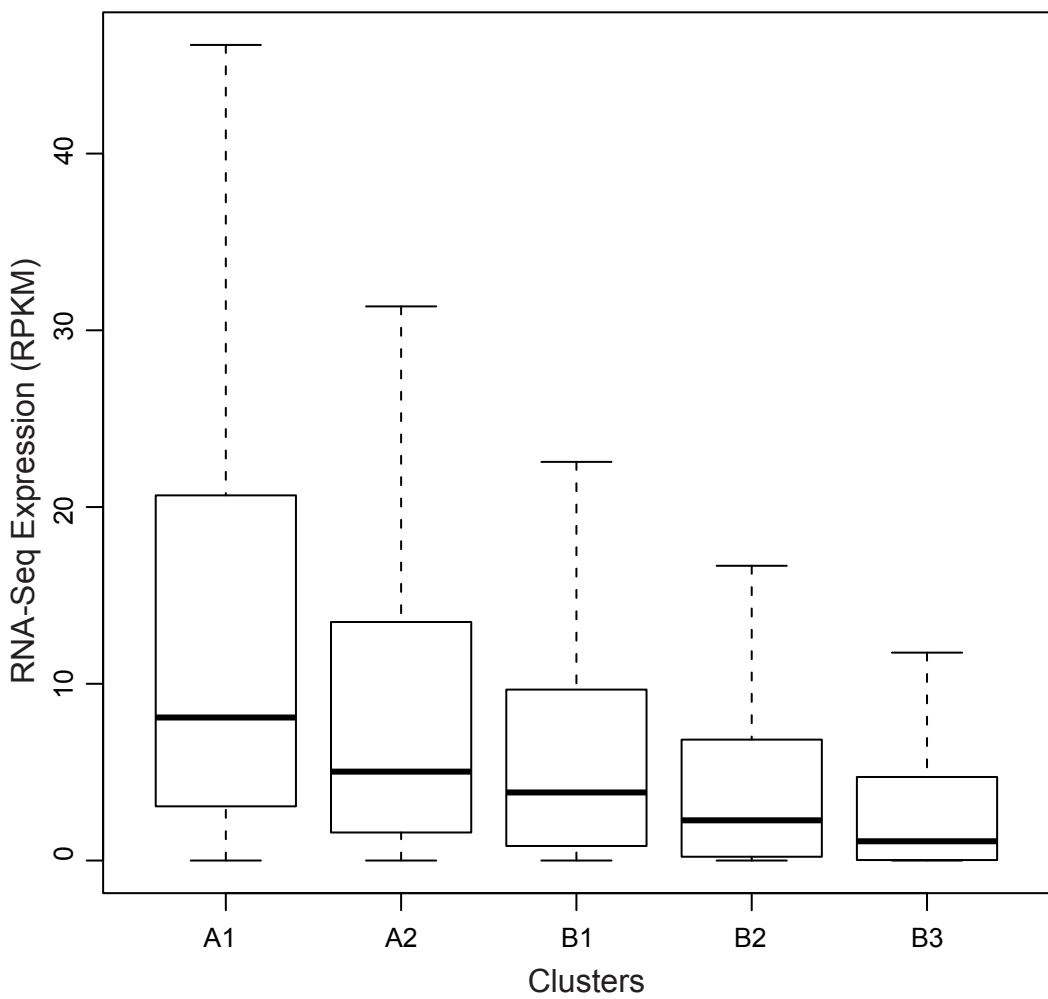

Supplement: Supplementary file 4 — Additional file 4: Figure S3: Chromatin profiles are associated with gene expression. p63 bound locations are clustered by 5 active (H3K4me1, H3K4me2, H3K4me3, H3K9ac, H3K27ac) and 2 repressive (H3K9me3, H3K27me3) histone modifications (Figure 2). The 5 groups (A1, A2, B1, B2, B3) of p63 targets are annotated to the nearest genes by GREAT. Box plots of gene expression as determined by RNA-Seq in keratinocytes, is shown. (PDF 82 KB) [file 12864_2014_6850_MOESM4_ESM.pdf]
