# Supplementary material for: Role of chromatin and transcriptional co-regulators in mediating p63-genome interactions in keratinocytes
Source: BMC Genomics. 2014 Nov 29;15(1):1042. doi: 10.1186/1471-2164-15-1042 (PMC4302094; doi:10.1186/1471-2164-15-1042)
Supplement: Supplementary file 5 — Additional file 5: Table S2: Discriminant Models. Fisher’s Discriminant models are made in SAS statistical software using DISCRIM procedure with 11 predictors. The full models use all the 10 chromatin marks. Significant chromatin marks models use 8 chromatin marks (Significant, if P value <0.0001). Regression 3 Model is made with the three marks (H3K4me1, DNase, DF) used to make the final regression model. Best 3 is the final discriminant model used to predict p63 binding, made with the three predictors having the highest predictive power (H3K4me1, DF, H3K27me3) according to STEPDISC procedure in SAS statistical software. Sensitivity and Specificity are used to judge the accuracy of the models. (PDF 411 KB) [file 12864_2014_6850_MOESM5_ESM.pdf]

# Table S2: Discriminant Models

| | Chromatin Marks | | | | | | | | | | | | Accuracy | |
|---|---|---|---|---|---|---|---|---|---|---|---|---|---|---|
| Model | H3K4me1 | H3K27me3 | DF (DNase-FAIRE interaction term) | FAIRE | H3K4me2 | H3K4me3 | H3K9ac | DNase | H3K27ac | H3K9me3 | Motif | Intercept | Sensitivity | Specificity |
| Best 3 | 30.96 | -5.99 | 13.95 | | | | | | | | | -25.09 | 89.08% | 97.65% |
| Best 3 + motif | 31.69 | -4.32 | 14.31 | | | | | | | | -0.04 | -24.59 | 88.82% | 97.64% |
| Regression 3 | 29.49 | | 11.08 | | | | | 1.64 | | | | -24.07 | 89.62% | 97.54% |
| Regression 3 + motif | 32.81 | | -27.92 | | | | | 27.81 | | | -0.17 | -30.38 | 89.74% | 97.23% |
| All significant chromatin marks | 28.4 | -7.72 | 13.47 | | 9.39 | 1.63 | | 20.39 | 5.84 | -8.76 | | -25.78 | 91.18% | 96.99% |
| All significant chromatin marks + motif | 29.77 | -5.95 | -28.61 | | 9.69 | 1.65 | | 30.37 | -5.79 | -5.06 | -0.11 | -29.85 | 90.95% | 96.90% |
| Full Discriminant Analysis model | 29.73 | -7.03 | -18.55 | -8.73 | 9.79 | 2.17 | -1.04 | 25.2 | -5.56 | -6.69 | | -25.65 | 90.84% | 97.00% |
| Full Discriminant Analysis model + motif | 29.89 | -5.91 | -28.76 | -1.57 | 9.73 | 2.1 | -0.9 | 30.73 | -5.47 | -5.02 | -0.1 | -29.54 | 90.90% | 96.93% |
