# Supplementary material for: Role of chromatin and transcriptional co-regulators in mediating p63-genome interactions in keratinocytes
Source: BMC Genomics. 2014 Nov 29;15(1):1042. doi: 10.1186/1471-2164-15-1042 (PMC4302094; doi:10.1186/1471-2164-15-1042)
Supplement: Supplementary file 6 — Additional file 6: Table S3: Regression Models. Regression models are made in SAS statistical software using REG procedure. The full models use all the 10 chromatin marks as predictors (Each predictor is significant at P-value 0.0001). Best 3 Model is made with the three marks (H3K4me1, DNase, DF) that together have the highest predictive power (Accounts for 74.8% variability in p63 binding, MSE = 0.08). Finally models with each individual predictor are also made. (PDF 885 KB) [file 12864_2014_6850_MOESM6_ESM.pdf]

Table S3: Regression Models

	Chromatin Marks												Accuracy			
**Model**	**H3K27me3**	**H3K9me3**	**H3K4me3**	**H3K9ac**	**FAIRE**	**H3K27ac**	**H3K4me2**	**H3K4me1**	**DNase**	**DF (DNase-FAIRE interaction term)**	**Motif**	**Intercept**	**R^2**	**MSE**	**F value**	**P-value**
**H3K27me3**	0.1827											0.3435	0.008	0.3709	453.73	3e-100
**H3K9me3**		0.4837										0.2688	0.020	0.3653	1166.256	<1e-200
**H3K4me3**			1.0664									0.1467	0.290	0.2471	24664.98	<1e-200
**H3K9ac**				1.0674								0.139	0.344	0.2322	31055.23	<1e-200
**FAIRE**					2.609							-0.277	0.427	0.1858	48288.28	<1e-200
**H3K27ac**						0.9162						0.1764	0.485	0.1872	54354.22	<1e-200
**H3K4me2**							1.1938					0.0932	0.572	0.1432	83889.03	<1e-200
**H3K4me1**								1.2704				0.071	0.655	0.1104	124600.5	<1e-200
**DNase**									1.593			-0.059	0.665	0.1144	122062.2	<1e-200
**DF (DNase-FAIRE interaction term)**										2.7255		0.1016	0.672	0.1163	121399.8	<1e-200
**Motif**											0.0151	0.4871	0.140	0.3096	9531.949	<1e-200
**Best 3**								0.6939	0.628	0.4981		-0.014	0.748	0.0828	63204.5	<1e-200
**Best 3 + motif**								0.682	0.416	0.8247	0.0016	0.0241	0.749	0.0826	47599.55	<1e-200
**Full regression model**	-0.0302	0.0237	-0.0707	-0.041	0.128	-0.089	0.213	0.5927	0.565	0.6438		-0.024	0.750	0.0817	19251.93	<1e-200
**Full Regression model + motif**	-0.0484	-0.0087	-0.0667	-0.04	-0.02	-0.094	0.2153	0.5965	0.449	0.8691	0.0022	0.0495	0.744	0.0815	17432.86	<1e-200
